# Supplementary material for: Seroprevalence and associated factors of HIV, syphilis, hepatitis B, and hepatitis C infections among sex workers in Chiangmai, Thailand during easing of COVID-19 lockdown measures
Source: PLoS One. 2024 Dec 31;19(12):e0316668. doi: 10.1371/journal.pone.0316668 (PMC11687872; doi:10.1371/journal.pone.0316668)
Supplement: S1 Table — (PDF) [file pone.0316668.s001.pdf]

S1 Table. Factors associated with HIV Ab positivity among male sex workers.

Characteristics		Male				
		n/N (%)	Univariable		Multivariable	
			OR (95%CI)	*p*-value	OR (95%CI)	*p*-value
Age (years)	≤ median age (27)	9/75 (12.0)	1.00			
	> median age (27)	9/63 (14.3)	1.22 (0.45-3.29)	0.692		
Race	Non-Thai	7/49 (14.3)	1.00			
	Thai	11/89 (12.4)	0.85 (0.31-2.34)	0.748		
Highest level of education	Lower than University/college	15/128 (11.7)	1.00			
	University/college	3/10 (30.0)	3.23 (0.75-13.84)	**0.115**		N.S.
Marital status	Single	16/98 (16.3)	1.00			
	Currently have a partner	2/29 (6.9)	0.38 (0.08-1.76)	**0.216**		N.S.
	Separated/divorced/widowed	0/11	N/A			
Have kids	No	14/99 (14.1)	1.00			
	Yes	4/39 (10.3)	0.69 (0.21-2.26)	0.543		
Smoking	No	9/45 (20.0)	1.00			
	Yes	9/93 (9.7)	0.43 (0.16-1.17)	**0.098**		N.S.
Drinking alcohol	No	4/20 (20.0)	1.00			
	Yes	14/118 (11.9)	0.54 (0.16-1.84)	0.324		
Recreational drug used, in the past 3 months	No	11/96 (11.5)	1.00			
	Yes	7/42 (16.7)	1.55 (0.55-4.31)	0.406		
Ever used drug injection	No	14/123 (11.4)	1.00			
	Yes	4/15 (26.7)	2.83 (0.79-10.11)	**0.109**		N.S.
Ever been diagnosed with genital infections	No	6/44 (13.6)	1.00			
	Yes	12/88 (13.6)	1.00 (0.35-2.87)	1.000		
Ever been diagnosed with HCV	No	14/102 (13.7)	1.00			
	Yes	1/2 (50.0)	6.29 (0.37-106.37)	**0.203**		N.S.
Ever had surgery or blood transfusion	No	16/99 (16.2)	1.00			
	Yes	2/38 (5.3)	0.29 (0.06-1.32)	**0.109**		N.S.
Ever had tattoos or piercing	No	3/24 (12.5)	1.00			

	Yes	15/114 (13.2)	1.06 (0.28-3.99)	0.931		
Ever visited a community barber for shaving (males)	No	5/70 (7.1)	1.00			
	Yes	13/68 (19.1)	3.07 (1.03-9.16)	**0.044**		N.S.
Sexual orientation	Heterosexual	0/4	N/A			
	Homosexual	5/15 (33.3)	4.08 (1.21-13.79)	**0.024**		N.S.
	Bisexual	13/119 (10.9)	1.00			
Age at first sexual intercourse	< 15 years old	6/43 (14.0)	1.00			
	> 15 years old	12/95 (12.6)	0.89 (0.31-2.56)	0.831		
Duration in sex work	< 2 years	2/50 (4.0)	1.00		1.00	
	> 2 years	16/88 (18.2)	5.33 (1.17-24.26)	**0.030**	3.97 (0.83-19.02)	0.084
Vaginal sex	No	4/15 (26.7)	1.00			
	Yes	14/123 (11.4)	0.35 (0.09-1.26)	**0.109**		N.S.
Receptive anal sex	No	10/107 (9.4)	1.00		1.00	
	Yes	8/31 (25.8)	3.37 (1.20-9.50)	**0.021**	3.68 (1.17-11.55)	**0.026**
Condom use with clients, in the past month	All the time	8/83 (9.6)	1.00		1.00	
	Never or occasionally	9/41 (22.0)	2.64 (0.93-7.45)	**0.067**	2.81 (0.92-8.56)	0.069
