# Supplementary material for: Seroprevalence and associated factors of HIV, syphilis, hepatitis B, and hepatitis C infections among sex workers in Chiangmai, Thailand during easing of COVID-19 lockdown measures
Source: PLoS One. 2024 Dec 31;19(12):e0316668. doi: 10.1371/journal.pone.0316668 (PMC11687872; doi:10.1371/journal.pone.0316668)
Supplement: S2 Table — (PDF) [file pone.0316668.s002.pdf]

S2 Table. Factors associated with *Treponema pallidum* Ab positivity among male sex workers.

**Characteristics**		**Male**				
		**n/N (%)**	**Univariable**		**Multivariable**	
			**OR (95%CI)**	***p*-value**	**OR (95%CI)**	***p*-value**
Age (years)	≤ median age (27)	21/75 (28.0)	1.00		1.00	
	> median age (27)	12/63 (19.1)	0.61 (0.27-1.35)	**0.222**	0.40 (0.13-1.29)	0.126
Race	Non-Thai	9/49 (18.4)	1.00			
	Thai	24/89 (27.0)	1.64 (0.69-3.88)	0.260		
Highest level of education	Lower than University/college	27/128 (21.1)	1.00			
	University/college	6/10 (60.0)	5.61 (1.47-21.32)	**0.011**		N.S.
Marital status	Single	26/98 (26.5)	1.00			
	Has a partner	5/29 (17.2)	0.58 (0.20-1.67)	0.310		
	Separated/divorced/widowed	2/11 (18.2)	0.62 (0.12-3.04)	0.551		
Have kids	No	28/99 (28.3)	1.00			
	Yes	5/39 (12.8)	0.37 (0.13-1.05)	**0.062**		N.S.
Smoking	No	8/45 (17.8)	1.00			
	Yes	25/93 (26.9)	1.70 (0.70-4.15)	**0.243**		N.S.
Drinking alcohol	No	7/20 (35.0)	1.00			
	Yes	26/118 (22.0)	0.52 (0.19-1.45)	**0.214**		N.S.
Recreational drug used, in the past 3 months	No	18/96 (18.8)	1.00		1.00	
	Yes	15/42 (35.7)	2.41 (1.07-5.43)	**0.034**	4.04 (1.33-12.27)	**0.014**
Ever used drug injection	No	28/123 (22.8)	1.00			
	Yes	5/15 (33.3)	1.70 (0.53-5.38)	0.369		
Ever been diagnosed with genital infections	No	5/44 (11.4)	1.00		1.00	
	Yes	27/88 (30.7)	3.45 (1.23-9.72)	**0.019**	3.28 (0.86-12.54)	0.083
Sexual orientation	Heterosexual	0/4	N/A			
	Homosexual	8/15 (53.3)	4.30 (1.42-12.99)	**0.010**		N.S.
	Bisexual	25/119 (21.0)	1.00			
Age at first sexual intercourse	< 15 years old	16/43 (37.2)	1.00		1.00	
	> 15 years old	17/95 (17.9)	0.37 (0.16-0.83)	**0.016**	0.37 (0.13-1.08)	0.069

Duration in sex work	< 2 years	10/50 (20.0)	1.00			
	> 2 years	23/88 (26.1)	1.42 (0.61-3.28)	0.418		
Vaginal sex	No	6/15 (40.0)	1.00			
	Yes	27/123 (21.9)	0.42 (0.14-1.29)	**0.130**		N.S.
Receptive anal sex	No	18/107 (16.8)	1.00		1.00	
	Yes	15/31 (48.4)	4.64 (1.95-11.04)	**0.001**	15.49 (4.31-55.65)	**0.000**
Oral sex	No	2/20 (10.0)	1.00			
	Yes	31/118 (26.3)	3.21 (0.70-14.62)	**0.132**		N.S.
Using sex toys	No	23/109 (21.1)	1.00			
	Yes	10/29 (34.5)	1.97 (0.81-4.81)	**0.137**		N.S.
Condom use with clients, in the past month	All the time	14/83 (16.9)	1.00			
	Never or occasionally	14/41 (34.2)	2.56 (1.08-6.06)	**0.033**		N.S.
