# Supplementary material for: Seroprevalence and associated factors of HIV, syphilis, hepatitis B, and hepatitis C infections among sex workers in Chiangmai, Thailand during easing of COVID-19 lockdown measures
Source: PLoS One. 2024 Dec 31;19(12):e0316668. doi: 10.1371/journal.pone.0316668 (PMC11687872; doi:10.1371/journal.pone.0316668)
Supplement: S3 Table — (PDF) [file pone.0316668.s003.pdf]

S3 Table. Factors associated with HBsAg positivity among male sex workers.

Characteristics		Male				
		n/N (%)	Univariable		Multivariable	
			OR (95%CI)	*p*-value	OR (95%CI)	*p*-value
Age	≤ 30 years	4/98 (4.1)	1.00			
	> 30 years	2/40 (5.0)	1.24 (0.22-7.04)	0.811		
Race	Non-Thai	5/49 (10.2)	1.00		1.00	
	Thai	1/89 (1.1)	0.1 (0.01-0.88)	**0.038**	0.14 (0.01-1.35)	0.089
Ever visited a community barber for shaving (males)	No	5/70 (7.1)	1.00			
	Yes	1/68 (1.5)	0.19 (0.02-1.71)	**0.139**		N.S.
Sexual orientation	Heterosexual	2/4 (50.0)	1.00		1.00	
	Homosexual	0/15	N/A			
	Bisexual	4/119 (3.4)	0.03 (0-0.31)	**0.003**	0.05 (0-0.53)	**0.012**
Age at first sexual intercourse	< 15 years old	1/43 (2.3)	1.00			
	> 15 years old	5/95 (5.3)	2.33 (0.26-20.60)	0.446		
Duration in sex work	< 2 years	3/50 (6.0)	1.00			
	> 2 years	3/88 (3.4)	0.55 (0.11-2.85)	0.479		
