# Supplementary material for: Seroprevalence and associated factors of HIV, syphilis, hepatitis B, and hepatitis C infections among sex workers in Chiangmai, Thailand during easing of COVID-19 lockdown measures
Source: PLoS One. 2024 Dec 31;19(12):e0316668. doi: 10.1371/journal.pone.0316668 (PMC11687872; doi:10.1371/journal.pone.0316668)
Supplement: S4 Table — (PDF) [file pone.0316668.s004.pdf]

S4 Table. Factors associated with HCV Ab positivity among male sex workers.

Characteristics		Male				
		n/N (%)	Univariable		Multivariable	
			OR (95%CI)	*p*-value	OR (95%CI)	*p*-value
Age (years)	≤ median age (27)	1/75 (1.4)	1.00		1.00	
	> median age (27)	8/63 (12.7)	10.76 (1.31-88.60)	**0.027**	15.12 (1.31-174.24)	**0.029**
Race	Non-Thai	4/49 (8.2)	1.00			
	Thai	5/89 (5.6)	0.67 (0.17-2.62)	0.564		
Drinking alcohol	No	3/20 (15.0)	1.00			
	Yes	6/118 (5.1)	0.30 (0.07-1.33)	**0.114**		N.S.
Recreational drug used, in the past 3 months	No	2/96 (2.1)	1.00		1.00	
	Yes	7/42 (16.7)	9.40 (1.86-47.44)	**0.007**	11.99 (1.67-86.12)	**0.014**
Ever used drug injection	No	3/123 (2.4)	1.00		1.00	
	Yes	6/15 (40.0)	26.67 (5.70-124.71)	**0.000**	21.89 (3.45-138.79)	**0.001**
Ever been diagnosed with genital infections	No	2/44 (4.6)	1.00			
	Yes	7/88 (8.0)	1.81 (0.36-9.13)	0.470		
Ever had shared sharp objects with others	No	3/73 (4.1)	1.00			
	Yes	6/65 (9.2)	2.37 (0.57-9.90)	**0.236**		N.S.
Ever had tattoos or piercing	No	1/24 (4.2)	1.00			
	Yes	8/114 (7.0)	1.74 (0.21-14.57)	0.611		
Sexual orientation	Heterosexual	0/4	N/A			
	Homosexual	2/15 (13.3)	2.46 (0.46-13.12)	0.291		
	Bisexual	7/119 (5.9)	1.00			
Age at first sexual intercourse	< 15 years old	4/43 (9.3)	1.00			
	> 15 years old	5/95 (5.3)	0.54 (0.14-2.13)	0.380		
Duration in sex work	< 2 years	1/50 (2.0)	1.00			
	> 2 years	8/88 (9.1)	4.9 (0.59-40.38)	**0.140**		N.S.
Receptive anal sex	No	5/107 (4.7)	1.00			
	Yes	4/31 (12.9)	3.02 (0.76-12.03)	**0.117**		N.S.
Using sex toys	No	5/109 (4.6)	1.00			
	Yes	4/29 (13.8)	3.33 (0.83-13.30)	**0.089**		N.S.
