# Supplementary material for: Seroprevalence and associated factors of HIV, syphilis, hepatitis B, and hepatitis C infections among sex workers in Chiangmai, Thailand during easing of COVID-19 lockdown measures
Source: PLoS One. 2024 Dec 31;19(12):e0316668. doi: 10.1371/journal.pone.0316668 (PMC11687872; doi:10.1371/journal.pone.0316668)
Supplement: S5 Table — (PDF) [file pone.0316668.s005.pdf]

S5 Table. Factors associated with HIV Ab positivity among female sex workers.

**Characteristics**			**Female**			
		**n/N (%)**	**Univariable**		**Multivariable**	
			**OR (95%CI)**	***p*-value**	**OR (95%CI)**	***p*-value**
Age (years)	$\leq$ median age (35.5)	1/63 (1.6)	1.00			
	> median age (35.5)	5/63 (7.9)	5.34 (0.61-47.13)	**0.131**		N.S.
Race	Non-Thai	1/21 (4.8)	1.00			
	Thai	5/105 (4.8)	1.00 (0.11-9.03)	1.000		
Highest level of education	Lower than University/college	5/112 (4.5)	1.00			
	University/college	1/14 (7.1)	1.65 (0.18-15.20)	0.660		
Marital status	Single	4/82 (4.9)	1.00			
	Has a partner	1/24 (4.2)	0.85 (0.09-7.96)	0.885		
	Separated/divorced/widowed	1/20 (5.0)	1.03 (0.11-9.72)	0.982		
Have kids	No	2/38 (5.3)	1.00			
	Yes	4/88 (4.6)	0.86 (0.15-4.89)	0.862		
Monthly income	<15,000 Baht	5/57 (8.8)	1.00			
	>15,000 Baht	1/69 (1.5)	0.15 (0.02-1.35)	**0.091**		N.S.
Smoking	No	3/90 (3.3)	1.00			
	Yes	3/36 (8.3)	2.64 (0.51-13.72)	**0.249**		N.S.
Drinking alcohol	No	3/20 (15.0)	1.00		1.00	
	Yes	3/106 (2.8)	0.17 (0.03-0.89)	**0.036**	0.17 (0.03-1.15)	0.070
Recreational drug used, in the past 3 months	No	4/113 (3.5)	1.00			
	Yes	2/13 (15.4)	4.95 (0.81-30.19)	**0.083**		N.S.
Ever been diagnosed with genital infections	No	4/92 (4.4)	1.00			
	Yes	1/21 (4.8)	1.10 (0.12-10.38)	0.934		
Ever had surgery or blood transfusion	No	2/84 (2.4)	1.00			
	Yes	4/41 (9.8)	4.43 (0.78-25.28)	**0.094**		N.S.
Sexual orientation	Heterosexual	4/115 (3.5)	1.00			
	Homosexual	0/1	N/A			
	Bisexual	2/10 (20.0)	6.94 (1.10-43.80)	**0.039**		N.S.

Age at first sexual intercourse	< 15 years old	3/14 (21.4)	1.00		1.00	
	> 15 years old	3/112 (2.7)	0.10 (0.02-0.56)	**0.009**	0.08 (0.01-0.59)	**0.013**
Duration in sex work	< 2 years	1/28 (3.6)	1.00			
	> 2 years	5/98 (5.1)	1.45 (0.16-12.96)	0.739		
Receptive anal sex	No	4/106 (3.8)	1.00			
	Yes	2/19 (10.5)	3.00 (0.51-17.67)	**0.225**		N.S.
Using sex toys	No	3/104 (2.9)	1.00		1.00	
	Yes	3/22 (13.6)	5.32 (1.00-28.34)	**0.050**	7.37 (1.00-53.85)	**0.049**
Condom use with clients, in the past month	All the time	4/103 (3.9)	1.00			
	Never or occasionally	2/19 (10.5)	2.91 (0.49-17.16)	**0.238**		N.S.
