# Supplementary material for: Seroprevalence and associated factors of HIV, syphilis, hepatitis B, and hepatitis C infections among sex workers in Chiangmai, Thailand during easing of COVID-19 lockdown measures
Source: PLoS One. 2024 Dec 31;19(12):e0316668. doi: 10.1371/journal.pone.0316668 (PMC11687872; doi:10.1371/journal.pone.0316668)
Supplement: S6 Table — (PDF) [file pone.0316668.s006.pdf]

S6 Table. Factors associated with *Treponema pallidum* Ab positivity among female sex workers.

Characteristics		Female				
		n/N (%)	Univariable		Multivariable	
			OR (95%CI)	*p*-value	OR (95%CI)	*p*-value
Age (years)	≤ median age (35.5)	5/63 (7.9)	1.00			
	> median age (35.5)	3/63 (4.8)	0.58 (0.13-2.54)	0.470		
Race	Non-Thai	2/21 (9.5)	1.00			
	Thai	6/105 (5.7)	0.58 (0.11-3.07)	0.518		
Highest level of education	Lower than University/college	7/112 (6.3)	1.00			
	University/college	1/14 (7.1)	1.15 (0.13-10.14)	0.897		
Marital status	Single	6/82 (7.3)	1.00			
	Has a partner	2/24 (8.3)	1.15 (0.22-6.11)	0.868		
	Separated/divorced/widowed	0/20	N/A			
Have kids	No	0/38	N/A			
	Yes	8/88 (9.1)				
Smoking	No	3/90 (3.3)	1.00		1.00	
	Yes	5/36 (13.9)	4.68 (1.06-20.73)	**0.042**	8.86 (1.59-49.26)	**0.013**
Drinking alcohol	No	0/20	N/A			
	Yes	8/106 (7.6)				
Recreational drug used, in the past 3 months	No	7/113 (6.2)	1.00			
	Yes	1/13 (7.7)	1.26 (0.14-11.15)	0.834		
Ever used drug injection	No	8/125 (6.4)				
	Yes	0/1	N/A			
Ever been diagnosed with genital infections	No	5/92 (5.4)	1.00			
	Yes	1/21 (4.8)	0.87 (0.09-7.86)	0.901		
Sexual orientation	Heterosexual	8/115 (7.0)				
	Homosexual	0/1	N/A			
	Bisexual	0/10	N/A			
Age at first sexual intercourse	< 15 years old	2/14 (14.3)	1.00			
	> 15 years old	6/112 (5.4)	0.34 (0.06-1.87)	**0.215**		N.S.

Duration in sex work	< 2 years	2/28 (7.1)	1.00			
	> 2 years	6/98 (6.1)	0.85 (0.16-4.45)	0.845		
Receptive anal sex	No	5/106 (4.7)	1.00		1.00	
	Yes	3/19 (15.8)	3.79 (0.82-17.41)	**0.087**	14.51 (1.88-111.76)	**0.010**
Oral sex	No	3/17 (17.7)	1.00		1.00	
	Yes	5/109 (4.6)	0.22 (0.05-1.04)	**0.057**	0.06 (0.01-0.48)	**0.008**
Condom use with clients, in the past month	All the time	5/103 (4.9)	1.00			
	Never or occasionally	3/19 (15.8)	3.68 (0.80-16.90)	**0.095**		N.S.
