# Supplementary material for: Seroprevalence and associated factors of HIV, syphilis, hepatitis B, and hepatitis C infections among sex workers in Chiangmai, Thailand during easing of COVID-19 lockdown measures
Source: PLoS One. 2024 Dec 31;19(12):e0316668. doi: 10.1371/journal.pone.0316668 (PMC11687872; doi:10.1371/journal.pone.0316668)
Supplement: S7 Table — (PDF) [file pone.0316668.s007.pdf]

**S7 Table. Factors associated with HBsAg positivity among female sex workers.**

| **Characteristics** | | **Female** | | | | |
|---|---|---|---|---|---|---|
| | | **n/N (%)** | **Univariable** | | **Multivariable** | |
| | | | **OR (95%CI)** | ***p*-value** | **OR (95%CI)** | ***p*-value** |
| Age | ≤ 30 years | 0/29 | N/A | | | |
| | > 30 years | 12/97 (12.4) | | | | |
| Race | Non-Thai | 2/21 (9.5) | 1.00 | | | |
| | Thai | 10/105 (9.5) | 1.00 (0.20-4.93) | 1.000 | | |
| Marital status | Single | 6/82 (7.3) | 1.00 | | 1.00 | |
| | Has a partner | 1/24 (4.2) | 0.55 (0.06-4.81) | 0.590 | | |
| | Separated/divorced/widowed | 5/20 (25.0) | 4.22 (1.14-15.64) | **0.031** | 4.37 (1.20-15.60) | **0.025** |
| Ever had surgery or blood transfusion | No | 6/84 (7.1) | 1.00 | | | |
| | Yes | 6/41 (14.6) | 2.23 (0.67-7.40) | **0.191** | | |
| Sexual orientation | Heterosexual | 11/115 (9.6) | 1.00 | | | |
| | Homosexual | 0/1 | N/A | | | |
| | Bisexual | 1/10 (10.0) | 1.05 (0.12-9.09) | 0.964 | | |
| Age at first sexual intercourse | < 15 years old | 1/14 (7.1) | 1.00 | | | |
| | > 15 years old | 11/112 (9.8) | 1.42 (0.17-11.88) | 0.749 | | |
| Duration in sex work | < 2 years | 1/28 (3.6) | 1.00 | | | |
| | > 2 years | 11/98 (11.2) | 3.41 (0.42-27.66) | **0.250** | | N.S. |
| Using sex toys | No | 8/104 (7.7) | 1.00 | | | |
| | Yes | 4/22 (18.2) | 2.67 (0.73-9.80) | **0.140** | | N.S. |
| Condom use with clients, in the past month | All the time | 8/103 (7.8) | 1.00 | | 1.00 | |
| | Never or occasionally | 4/19 (21.1) | 3.17 (0.85-11.83) | **0.087** | 3.03 (0.77-11.89) | 0.112 |
