# Supplementary material for: Seroprevalence and associated factors of HIV, syphilis, hepatitis B, and hepatitis C infections among sex workers in Chiangmai, Thailand during easing of COVID-19 lockdown measures
Source: PLoS One. 2024 Dec 31;19(12):e0316668. doi: 10.1371/journal.pone.0316668 (PMC11687872; doi:10.1371/journal.pone.0316668)
Supplement: S8 Table — (PDF) [file pone.0316668.s008.pdf]

S8 Table. Factors associated with HCV Ab positivity among female sex workers.

Characteristics		Female				
		n/N (%)	Univariable		Multivariable	
			OR (95%CI)	*p*-value	OR (95%CI)	*p*-value
Age (years)	$\leq$ median age (35.5)	2/63 (3.2)	1.00			
	> median age (35.5)	1/63 (1.6)	0.49 (0.04-5.57)	0.567		
Race	Non-Thai	1/21 (4.8)	1.00			
	Thai	2/105 (1.9)	0.39 (0-4.50)	0.449		
Recreational drug used, in the past 3 months	No	1/113 (0.9)	1.00			
	Yes	2/13 (15.4)	20.36 (1.71-242.94)	**0.017**		N.S.
Ever been diagnosed with genital infections	No	2/92 (2.2)	1.00			
	Yes	1/21 (4.8)	2.25 (0.19-26.04)	0.516		
Ever had surgery or blood transfusion	No	1/84 (1.2)	1.00			
	Yes	2/41 (4.9)	4.26 (0.37-48.37)	**0.243**		N.S.
Ever had tattoos or piercing	No	0/15	N/A			
	Yes	3/111 (2.7)				
Sexual orientation	Heterosexual	2/115 (1.7)	1.00			
	Homosexual	0/1	N/A			
	Bisexual	1/10 (10.0)	6.28 (0.52-76.07)	**0.149**		N.S.
Age at first sexual intercourse	< 15 years old	1/14 (7.1)	1.00			
	> 15 years old	2/112 (1.8)	0.24 (0.02-2.79)	0.252		
Duration in sex work	< 2 years	1/28 (3.6)	1.00			
	> 2 years	2/98 (2.0)	0.56 (0.05-6.44)	0.644		
Receptive anal sex	No	1/106 (0.9)	1.00			
	Yes	1/19 (5.3)	5.83 (0.35-97.53)	**0.220**		N.S.
