# Supplementary material for: Seroprevalence and associated factors of HIV, syphilis, hepatitis B, and hepatitis C infections among sex workers in Chiangmai, Thailand during easing of COVID-19 lockdown measures
Source: PLoS One. 2024 Dec 31;19(12):e0316668. doi: 10.1371/journal.pone.0316668 (PMC11687872; doi:10.1371/journal.pone.0316668)
Supplement: Attachment — Submitted filename: Responses to reviewer 2.docx [file pone.0316668.s009.docx]

**Response to Reviewer 2 comments**

Thank you very much for taking the time to review this manuscript and for giving us the opportunity to clarify and improve our manuscript. Please find below the detailed responses and the corresponding revisions/corrections highlighted in the re-submitted manuscript.

**Reviewer #2:** The authors estimated the seroprevalence of several STIs among sexual workers in Thailand by recruiting participants during COVID-19. The authors also assessed the potential factors that were linked to a higher seroprevalence of STIs. Below are some suggestions to help improve the content and scientific presentation of the study.

1. Lines 91-93. The recruitment period was from 1 Mar 2022 to 31 Dec 2022. “In this period, lockdown and confinement measures were being lifted, but night entertainment venues were still closed until June 2022.” The information referred to the situation that no lockdown and confinement measures throughout the recruitment period while night entertainment venues were still closed until June 2022. That is, the closure of “night entertainment venues” was the only measure relating to COVID-19 during the recruitment period. In this case, the authors are suggested to compare and/or discuss the seroprevalence of STIs and the associated factors before and after night entertainment venues were “resumed”. This is to respond to the title for the changes “during easing of COVID lockdown measures”.

**Response:** Thank you for your insightful comment regarding the comparison of seroprevalence of STIs and associated factors before and after the reopening of night entertainment venues in June 2022. We agree that this is an important context to consider in understanding the impact of COVID-19-related measures on STI dynamics. However, we respectfully note that the serological biomarkers assessed in our study reflect cumulative exposure to the pathogens over time, rather than acute or recent infections. Consequently, these biomarkers are less sensitive to short-term changes in behaviors that may have occurred due to the reopening of night entertainment venues during the recruitment period.

Furthermore, analyzing the data separately before and after June 2022 would require stratification based on a measure (i.e., reopening of venues) that may not directly influence the presence of these serological markers. For this reason, we chose to analyze the data for the entire recruitment period to provide a comprehensive assessment of the seroprevalence of STIs and their associated factors.

2. Please include sample size estimation. If no sample size estimation was done before the study, please justify or provide the reason.

**Response:** The sample size was calculated based on number of sex workers who living in Chiangmai (3,100 female sex worker and 672 male sex workers). Sample size was determined using Krejcie and Morgan formula. After calculation, 152 males and 185 females should be included in the study. During the data collection, the recruitment of participants was slow and challenging due to the closure of several entertainment venues in response to national restrictions during the COVID-19 pandemic. However, we believe that the sample size of 264 participants is sufficient to provide a representative seroprevalence estimate for the targeted population.

3. Lines 139-142. Multivariable logistic regression analyses.
3.a. Cite the reference(s) and the justification for using p at 0.250 as the threshold to pick variables in the multivariable model.

**Response:** Using a p-value threshold of <0.25 in univariate analysis to select variables for inclusion in multivariable logistic regression is a practice in statistical modeling. The rationale behind this approach is to avoid prematurely excluding variables that may not show strong statistical significance in univariate analysis but could become important predictors when adjusted for other covariates in the multivariable model.

Previous studies also suggested that variables with a p-value <0.25 in univariate screening should be considered for multivariable analysis to avoid overlooking important predictors [1-3].

References

1.	Bendel RB, Afifi AA. Comparison of Stopping Rules in Forward “Stepwise” Regression. Journal of the American Statistical Association. 1977;72(357):46-53. doi: 10.1080/01621459.1977.10479905.

2.	Mickey RM, Greenland S. The impact of confounder selection criteria on effect estimation. Am J Epidemiol. 1989;129(1):125-37. doi: 10.1093/oxfordjournals.aje.a115101. PMID: 2910056.

3.	Zhang Z. Model building strategy for logistic regression: purposeful selection. Annals of Translational Medicine. 2016;4(6):111.

We have added into the revised manuscript with the following sentences.

“Variables significant in the univariable analysis with a p-value of <0.250 were included in the multivariable logistic regression analysis. A cutoff value of 0.250 is supported by previous study [10].” (page 7, line 147-149)

3.b. “Variables that were not statistically significant with a p-value of >0.250 were removed from the model using a forward selection method.” Please elaborate on how to “remove” a variable when using a “forward selection method”.

**Response:** In the forward selection, we start with no variables and add them one by one, beginning with the one most correlated with the outcome. Variables considered more important are added first and remain in the model.

We have added into the revised manuscript with the following sentences.

“Variables that were not statistically significant with a p-value of >0.250 were removed from the model using a forward selection method, starting with no variables and adding them one by one, beginning with the one most correlated with the outcome.” (page 7, line 149-152)

4.a. Please change “p=0.000” to “p<0.001” throughout the text and the table.

**Response:** We have changed from “p=0.000” to “p<0.001” throughout the text and the table. (Table 1, line 183, Table 2, line 202, and Table 3)

4.b. Be consistence about the decimal places used for p-values. P-values for seroprevalence comparison on lines 166-177 were based on 3 decimal places while only 2 decimal places were presented for p-values for factor associations on lines 183-213.

**Response:** We have changed the decimal places used for p-values to 3 decimal places throughout the text and the table. (Table 1, line 201-207, Table 3, line 218-221, and Table 4)

5. Table 1. Include the p-value for comparing each characteristic between males and females, using the appropriate statistical tests. State the statistical tests that will be used in “Data analysis”.

**Response:** We have added the p-value for comparing each characteristic between males and females in Table 1 (page 8-10). We have also stated the statistical tests into the revised manuscript in the data analysis part with the following sentences.

“The comparison of characteristics between males and females was conducted using the Chi-square test for categorical variables and the Mann-Whitney U test for continuous variables.” (page 7, line 141-143)

6. Table 2. Please indicate whether the p-values referred to the comparison of seroprevalence between males and females. Include the total number of participants of each group (Total, Male, and Female) in the header row.

**Response:** We have modified Table 2 as suggested. (page 11)

7. Please explain or justify the thresholds used to categorize continuous variables into discrete groups in the regression analyses. For example, in lines 266-267, the authors discussed that male sexual workers who were over 27 years old were more likely to be HCV Ab positive. Why did they use 27 years old at the threshold/cutoff but not 30 or 25?

**Response:** Thank you very much for pointing this out. In response to your specific question regarding the threshold of 27 years old used to categorize age, we chose this value based on the median age of male participants in our study. Using the median age as a cutoff allows for a clear and balanced categorization of participants into two groups for comparative analysis. This approach minimizes potential biases and ensures an equal distribution of the sample across age groups, thereby enhancing the robustness and interpretability of our regression model.

8. Please also convert or include the monetary values into USD.

**Response:** We have converted the monetary values into USD. (Table 1, page 9)
